# Involvement of Tn3 transposon in formation and transmission of hypervirulent and carbapenem-resistant *Klebsiella pneumoniae*

**DOI:** 10.1128/spectrum.03038-23

**Published:** 2023-11-20

**Authors:** Dongxing Tian, Mingqi Zhao, Sasa Zheng, Xiaofei Jiang, Bin Zhang

**Affiliations:** 1 Department of Clinical Laboratory, Affiliated Hospital of Jining Medical University, Jining, Shandong Province, China; 2 Postdoctoral Mobile Station of Shandong University of Traditional Chinese Medicine, Jinan, Shandong Province, China; 3 Affiliated Hospital of Jining Medical University, Jining, Shandong Province, China; 4 Department of Oncology, Affiliated Hospital of Jining Medical University, Jining, Shandong Province, China; 5 Department of Clinical Laboratory, Huashan Hospital, Fudan University, Shanghai, China; Children's National Hospital, George Washington University, Washington, DC, USA

**Keywords:** Tn3 transposon, carbapenem resistance, virulence, horizontal gene transfer, *Klebsiella pneumoniae*

## Abstract

Hypervirulent and carbapenem-resistant *Klebsiella pneumoniae* poses a severe threat to public health for its high pathogenicity, transmissibility, and drug resistance. This study aims to explore the evolutionary path and the role of Tn3 transposon in the virulence and carbapenem resistance transmission of *K. pneumoniae*. Bioinformatics analysis and some experimental tests such as plasmid conjugation experiments, antimicrobial susceptibility testing, and virulence-associated tests were performed to explore the virulence and drug resistance transmission. The complete genome sequencing, S1 nuclease pulsed-field gel electrophoresis, and bioinformatics analysis were used to investigate their transmission mechanism mediated by Tn3 transposons. Three hypervirulent and carbapenem-resistant *K. pneumoniae* isolates, which were obtained from different patients at different time points in the same ward, harbored a virulence plasmid and a carbapenem resistance plasmid. They were confirmed to have first evolved from hypervirulent isolates and then acquired a *bla*_KPC_-positive plasmid (CR-hvKp evolutionary pattern). The non-conjugative virulence plasmid pVir could be transferred to other bacterial strains via mobilization by the conjugative IncN/U-type plasmid pKPC, as well as by fusing with the conjugative pKPC plasmid (mediated by Tn3-based homologous recombination) to be self-transmissible, thus transferring drug resistance and virulence. The cointegration, pVir/KPC fusion plasmid, was further resolved into pVir and pKPC between the duplicated copies of the Tn3 transposon resolution site mediated by site-specific recombination. Therefore, Tn3 transposons can mediate hypervirulent and carbapenem-resistant *K. pneumoniae* strains transferring drug resistance and virulence to other bacteria. We must be vigilant to emerging transposon-mediated hypervirulent and carbapenem-resistant pathogens.

**IMPORTANCE:**

Carbapenem-resistant *Klebsiella pneumoniae* (CRKP) is resistant to most common antibiotics, becoming the most important and prevalent nosocomial opportunity pathogen. Besides, *K. pneumoniae* can also cause severe community-acquired infections, such as primary liver abscess and endophthalmitis. These pathogens are commonly referred to as hvKp. CRKP and hvKp have evolved separately, each occupying its own clonal lineage and exhibiting a variety of properties. Our study provides important insights into the evolutionary events related to the arousal of virulence and drug resistance in *K. pneumoniae* through plasmid transmission, mediated by Tn3 transposon. Our study also provides evidence that multiple mechanisms contribute to the successful transfer of non-conjugative virulence plasmid, and the involvement of transposons enhances the efficiency. A good knowledge of its transmission mechanisms is fundamental to finding effective strategies to combat these threatening pathogens. Transposons are widely present in bacteria, spreading resistance and virulence genes between the environment and humans. Therefore, emerging transposon-mediated hypervirulent and carbapenem-resistant pathogens should be highly valued.

## INTRODUCTION

*Klebsiella pneumoniae* can survive in a wide range of ecological niches, either as a commensal microorganism or as an opportunistic pathogen. The rise of multi-drug resistance (MDR), particularly against the carbapenem antibiotic family, is an issue worthy of the highest clinical concern (1). *K. pneumoniae* can also be a threat outside of the hospital setting by causing severe community-acquired infections. A major characteristic of hypervirulent *K. pneumoniae* (hvKp) strains is their ability to cause invasive infections in multiple and unusual sites (2).

The phenotypes of MDR and hypervirulence are often thought to have evolved separately, each occupying its own clonal lineage and exhibiting a variety of properties (3, 4). The plasmids responsible for carbapenem resistance, which carry a type IV secretion system or another transfer element, can be transferred between *K. pneumoniae* strains via plasmid conjugation (5). Virulence plasmids can also be transferred between bacterial strains facilitated by other conjugative elements (6 – 8). Since both functions are mobile, genotypic convergence for carbapenem resistance and hypervirulence will be an outstanding issue to face, potentiating difficult-to-treat and invasive infections. Current boundaries between carbapenem-resistant *K. pneumoniae* (CRKP) and hvKp clones could be eroded, making *K. pneumoniae* a greater threat to public health. Several fatal outbreaks have been reported in China, where carbapenem-resistant and hypervirulent *K. pneumoniae* have been increasingly common (9 – 11).

The convergent events can be broadly divided into three pathways: acquisition of carbapenem resistance genes by hvKp clones (CR-hvKp); acquisition of virulence factors by CRKP clones (hv-CRKP); and the generation of hybrid vectors of both carbapenem resistance and virulence through genetic linkage of mobile genetic elements (MGEs) (12). Multiple mechanisms have been described for hybrid vectors, including the fusion of virulence and resistance plasmids (13), the insertion of antimicrobial resistance genes into common virulence plasmids, and the insertion of virulence regions into resistance plasmids (14). Transposons, one of the most common MGEs, allow the assembly, diversification, and redistribution of ever-growing arsenals of resistance and virulence genes. Transposons, along with other MGEs, contribute to the emergence of drug resistance and virulence at a rate that poses a challenge to new treatments (15, 16). Tn3 is a large and widespread transposon family whose role in the emergence and transmission of hypervirulent and carbapenem-resistant *K. pneumoniae* is largely unknown.

The most common working model of hypervirulent and carbapenem-resistant *K. pneumoniae* is likely the presence of virulence plasmids or hybrid plasmids in CRKP clones (hv-CRKP pattern) (6). ST11 hv-CRKP strains are disseminated in China, and multiple convergent strains have also been reported from South and Southeast Asia (17, 18). However, the acquisition of carbapenem resistance genes or plasmids by hvKp clones (CR-hvKp pattern) has been very rarely reported. Our previous study demonstrated that hvKp clones acquiring *bla*_KPC_-positive plasmids *in vitro* could not simultaneously exhibit carbapenem resistance and hypervirulence (6). However, limited information is available about the evolution and transmission of CR-hvKp strains directly isolated from patients.

In this study, we identified three ST268-hv-CRKP isolates harboring both the virulence-associated genes (*iutA-iucABCD*, *iroN-iroABCDE*, *rmpA*, and *rmpA2*) and the carbapenem resistance gene (*bla*_KPC-2_) in China. We will elaborate on the potential of ST268-K20 *K. pneumoniae* to develop into hypervirulent and carbapenem-resistant *K. pneumoniae* (CR-hvKp) and the essential role of Tn3 in the evolution and transmission of CR-hvKp strains. This study expands the function of Tn3 and advances our knowledge of the transmission mechanisms of hypervirulent and carbapenem-resistant *K. pneumoniae*, provides evidence on prophylactic strategies in the presence of CR-hvKp infections, and provides theoretical guidance for CR-hvKp infections.

## RESULTS

### Molecular characteristics of *K. pneumoniae* isolates

Between January 2017 and February 2018, a total of 530 multicenter clinical *K. pneumoniae* isolates were collected, including 34 isolates characterized as carbapenem-resistant and hypervirulent (6). Among these, three isolates (KP130, KP131, and KP133) were classified into the ST268-K20 type. They were positive both for specific virulence factors (*iucA, iroB, rmpA*, and *rmpA2*) and for the *bla*_KPC-2_ gene. This finding indicates that they simultaneously harbored the pK2044-like virulence plasmid and the KPC plasmid conferring hypervirulence and carbapenem resistance, respectively. Complete genomes of three CR-hvKp isolates were sequenced. Their genome characteristics are summarized in Table 1. Notably, the pKPC and pVir/KPC plasmids had complete conjugative transfer elements, indicating that they could be transferred to other strains and could even mobilize non-conjugative plasmids.

**TABLE 1:** The genomic characteristics of ST268-CR-hvKp strains in this study

Characteristics	KP130			KP131		KP133	
Chromosome (Kb)	5222751			5222700		5222700	
	pKP130-1	pKP130-2	pKP130-3	pKP131-1	pKP131-2	pKP133-1	pKP133-2
Accession no.	CP097083	CP097084	CP097085	CP097080	CP097081	CP097077	CP097078
Length (bp)	226,565	70,024	8,802	226,558	78,167	296,587	8802
GC content (%)	50.4	53.4	56.5	50.4	53.8	51.1	56.5
No. of ORF^*a*^	307	96	13	303	113	401	12
Incompatibility group	IncFIBK/IncHI1B	IncN/IncU	ColRNAI	IncFIBK/IncHI1B	IncN/IncU/ ColRNAI	IncFIBK/IncHI1B/IncN/IncU	ColRNAI
Conjugal transfer elements							
riT	+^*b*^	+	–	+	+	+	–
Relaxase	–^*c*^	+	–	–	+	+	–
T4CP	+	+	–	+	+	+	–
T4SS	–	+	–	–	+	+	–
Resistance genes	–	*bla*_KPC-2_ ; *arr-3; dfrA14; aac(6')-Ib10*	–	–	*bla*_KPC-2_ ; *arr-3; dfrA14; aac(6')-Ib10*	*bla*_KPC-2_ ; *arr-3; dfrA14; aac(6')-Ib10*	–
Virulence genes	*iucABCD-iutA*; *iroBCD-iroN rmpA rmpA2*	–	–	*iucABCD-iutA*; *iroBCD-iroN rmpA rmpA2*	–	*iucABCD-iutA*; *iroBCD-iroN rmpA rmpA2*	–

^*a*^ ORF, open reading frame.

^*b*^ +, have such information.

^*c*^ –, no such information.

### Evolutionary pathway of the ST268 CR-hvKp strains

According to the phylogenetic tree computed using the goeBURST software, ST268 belonged to the *K. pneumoniae* clonal group 815 (Group 2, Fig. S1A), adjacent to well-known MDR clones (Group 15; Fig. S1A), such as ST15 and ST14, and also adjacent to well-known hvKp clones (Group 35; Fig. S1A), such as CG23 and ST65. Group 1 had the highest resistance score, while Group 3 had the highest virulence score (Fig. S1B). The mean virulence and resistance scores of Group 2 were intermediate between Group 1 and Group 3 (Fig. S1B). From the bubble scatterplot data, it was evident that the ST268-adjacent genomes belonged to MDR clones (CG15) as well as hvKp clones (CG23 and ST65) (Fig. S1C). Therefore, the evolutionary pathway of the ST268 CR-hvKp strains is not straightforward to determine according to the goeBURST analysis because the ST268 clones were intermediate between hypervirulent and resistance clones.

**FIG 1:**
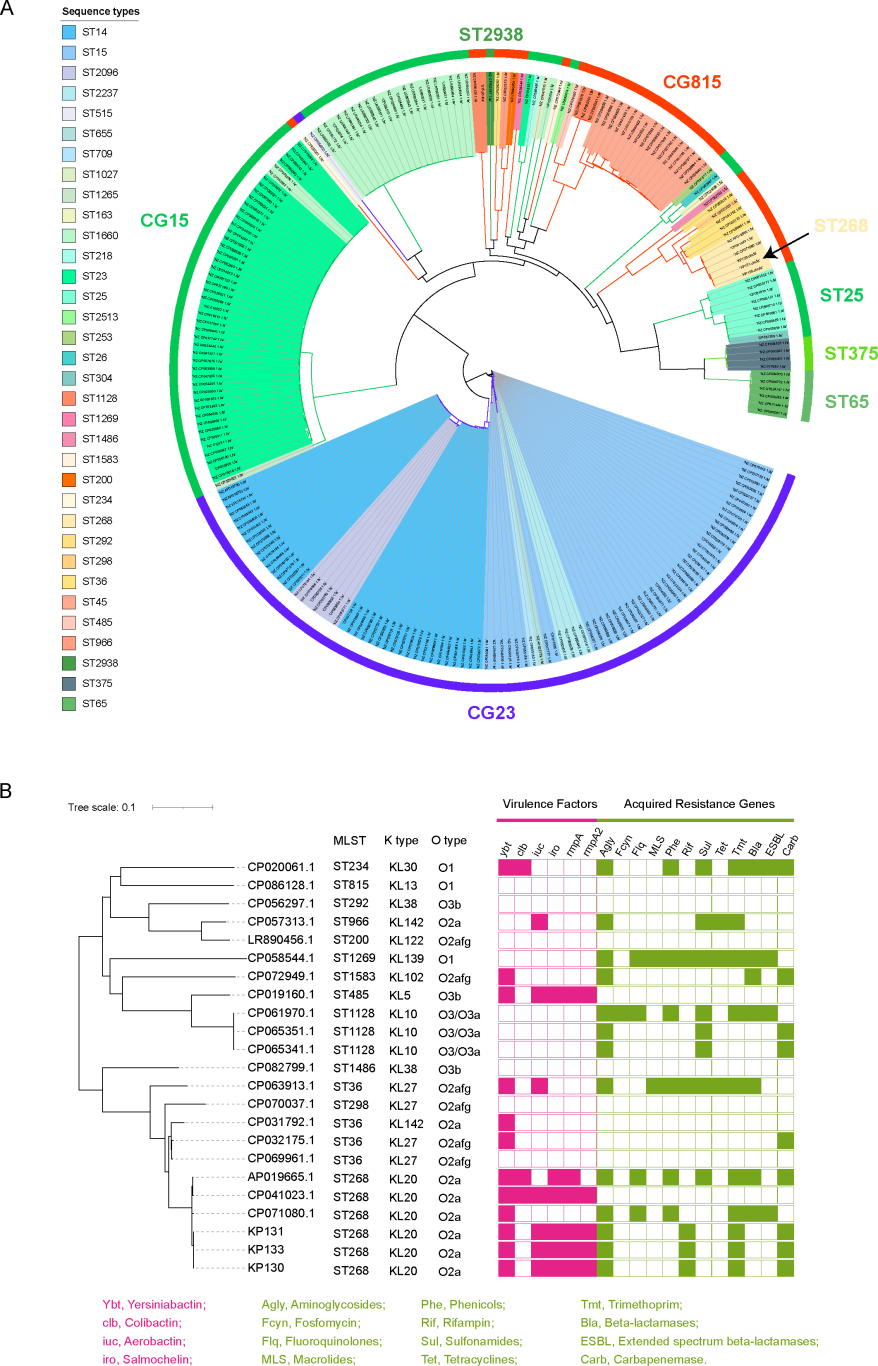
Genetic features of ST268 and other related *Klebsiella pneumoniae* genomes. (**A**) Phylogenetic tree based on core SNPs of 228 ST268-related *Klebsiella pneumoniae* genomes. (**B**) Characteristics of ST268 and adjacent genomes.

However, the single nucleotide polymorphism (SNP)-based phylogenetic tree of 228 genomes in this cluster showed that ST268 was closely related to KL2 hvKp clones, such as ST65, ST375, and ST25, and displayed a longer distance from CG23 hypervirulent and CG15 MDR clones (Fig. 1A). Three ST268 CR-hvKp strains in this study shared genetic similarities with other ST268 strains. The ST268 strains were more amenable to harbor resistance and virulence genes than other sequence types in Group 2 (Fig. 1B). Therefore, it is possible that the ST268 CR-hvKp strains first evolved from hypervirulent isolates and acquired KPC plasmids later.

### Phenotypic characteristics of resistance and virulence

The KP130, KP131, and KP133 *K. pneumoniae* strains had similar resistance phenotypes. They were resistant to cephems (cefazolin, cefuroxime, ceftriaxone, and cefepime), β-lactam combination agents (ampicillin/sulbactam, piperacillin/tazobactam, and cefoperazone/sulbactam), carbapenems (meropenem and imipenem), and were still susceptible to fluoroquinolones (levofloxacin), aminoglycosides (gentamicin and amikacin), nitrofurantoin, and tigecycline (Table 2). Thus, KP130, KP131, and KP133 belonged to a class of MDR clones.

**TABLE 2:** Antimicrobial susceptibilities of strains and their transconjugants

Strains	Bacterial species	MIC (µg/mL)^*a*^																
		AMP	SAM	TZP	CZO	CXM	CRO	FEP	CSL	CHL	GEN	LVX	SXT	IPM	MEM	TGC	NIT	AMK
KP130	*K. pneumoniae*	–^*b*^	>64/32	>256/4	>16	>64	>8	>64	64/32	8	≤1	≤0.5	>8/152	16	16	≤0.5	32	≤8
KP131	*K. pneumoniae*	–	>64/32	>256/4	>16	>64	>8	>64	128/64	4	≤1	≤0.5	>8/152	32	32	≤0.5	32	16
KP133	*K. pneumoniae*	–	>64/32	>256/4	>16	>64	>8	>64	64/32	32	≤1	≤0.5	>8/152	16	32	≤0.5	32	≤8
J53	*E. coli*	≤2	4/2	≤8/4	2	4	≤0.25	4	<4/2	8	≤1	≤0.5	≤0.25/4.75	≤0.25	≤0.25	≤0.5	16	≤8
Transconjugants																		
J53-p130	*E. coli*	>64	>64/32	>256/4	>16	>64	>8	64	64/32	8	2	≤0.5	4/76	1	2	≤0.5	≤8	≤8
J53-p131	*E. coli*	>64	>64/32	>256/4	>16	>64	>8	8	32/16	8	≤1	≤0.5	2/38	2	2	≤0.5	≤8	32
J53-p133	*E. coli*	>64	>64/32	>256/4	>16	>64	>8	>64	64/32	4	2	≤0.5	2/38	2	2	≤0.5	≤8	32

^*a*^ AMP, ampicillin; SAM, ampicillin/sulbactam; TZP, piperacillin/tazobactam; CZO, cefazolin; CXM, cefuroxime; CRO, ceftriaxone; FEP, cefepime; CSL, cefoperazone/sulbactam; CHL, chloramphenicol; GEN, gentamicin; LVX, levofloxacin; SXT, trimethoprim/sulphamethoxazole; IPM, imipenem; MEM, meropenem; TGC, tigecycline; NIT, nitrofurantoin; AMK, amikacin; *E. coli*, *Escherichia coli*.

^*b*^ Naturally resistant to ampicillin.

**FIG 2:**
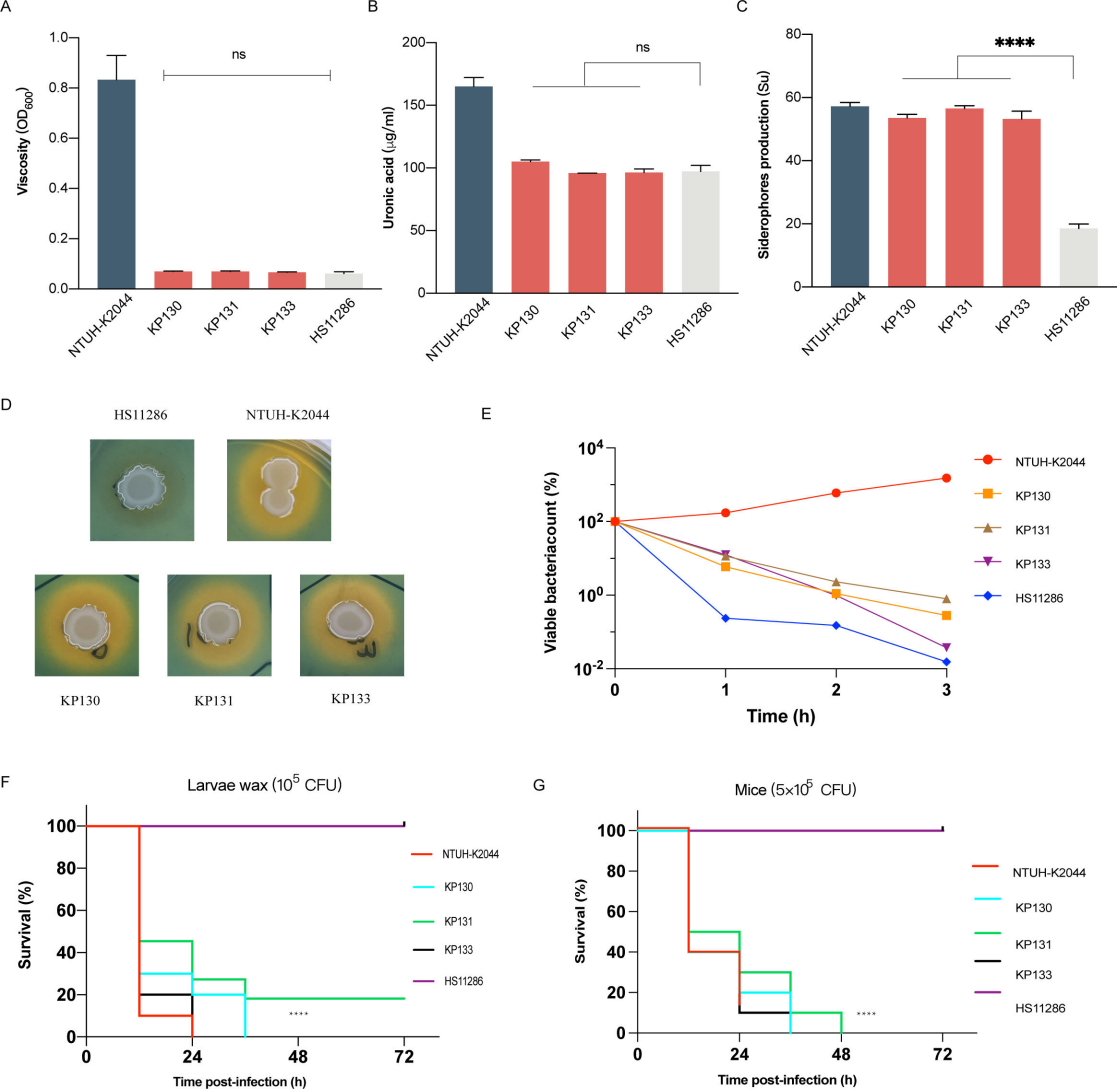
Virulence phenotypes and levels of ST268 isolates. (**A**) Hypermucoviscosity. (**B**) Capsular polysaccharide (CPS) production. (**C**) Quantification of siderophore production. (**D**) Siderophore production was determined by Chrome Azurol S agar plate. (**E**) Serum resistance. (**F**) Survival curves of infected larvae wax (1 × 10^5^ CFU). (**G**) Survival curves of infected mice (5 × 10^5^ CFU). An unpaired two-sided Student’s *t*-test was performed for mucoviscosity, uronic acid, and siderophores production analysis. A log-rank (Mantel–Cox) test was performed for the survival curves (****P < 0.0001, ns: not significant).

We observed that the KP130, KP131, and KP133 strains were all negative for the string test and clearly showed lower viscosity levels than the positive control strain NTUH-K2044 (ST23-K1; Fig. 2A). Furthermore, they did not produce high capsular polysaccharide (0) levels (Fig. 2B). They were also sensitive to human serum (Fig. 2E). However, we found that these strains had higher levels of siderophore production than those of ST23-K1 NTUH-K2044 (Fig. 2C and D). Moreover, the animal infection assays showed strong evidence of the hypervirulence of the KP130, KP131, and KP133 strains. The KP130, KP131, and KP133 strains exhibited significantly higher virulence levels, in comparison with the CRKP strain HS11286 (Fig. 2E and F). Taken together, KP130, KP131, and KP133 exhibited both carbapenem resistance and hypervirulence.

### Comparative genomic analysis of CR-hvKp plasmids

Although the KP130, KP131, and KP133 *K. pneumoniae* strains shared genetic similarities, they showed differences among the numbers and molecular weights of plasmids. KP130 had three plasmids: pKP130-1 (pVir, 226 Kb), pKP130-2 (pKPC, 70 Kb), and pKP130-3 (pCol, 8 Kb); KP131 had two plasmids, pKP131-1 (pVir, 226 Kb) and pKP131-2 (pKPC, 78 Kb); and KP133 had two plasmids, pKP133-1 (pVir/KPC, 296 Kb) and pKP133-2 (pCol, 8 Kb). The pVir plasmids of KP130 and KP131, which had virulence-associated genes, belonged to the IncFIB_K_/IncHI1B type. The pKPC plasmid of the KP130 and KP131 strains, which harbored the *bla*_KPC-2_ gene, belonged to the IncN/U type. Interestingly, the pKP133-1 plasmid was found to harbor both the virulence-associated genes and the *bla*_KPC_ gene, thus being classified into the pVir/KPC type. It is important to note that the *rmpA* (g.286C > G) and the *rmpA2* (g.56A > G) gene mutations were detected in these isolates.

**FIG 3:**
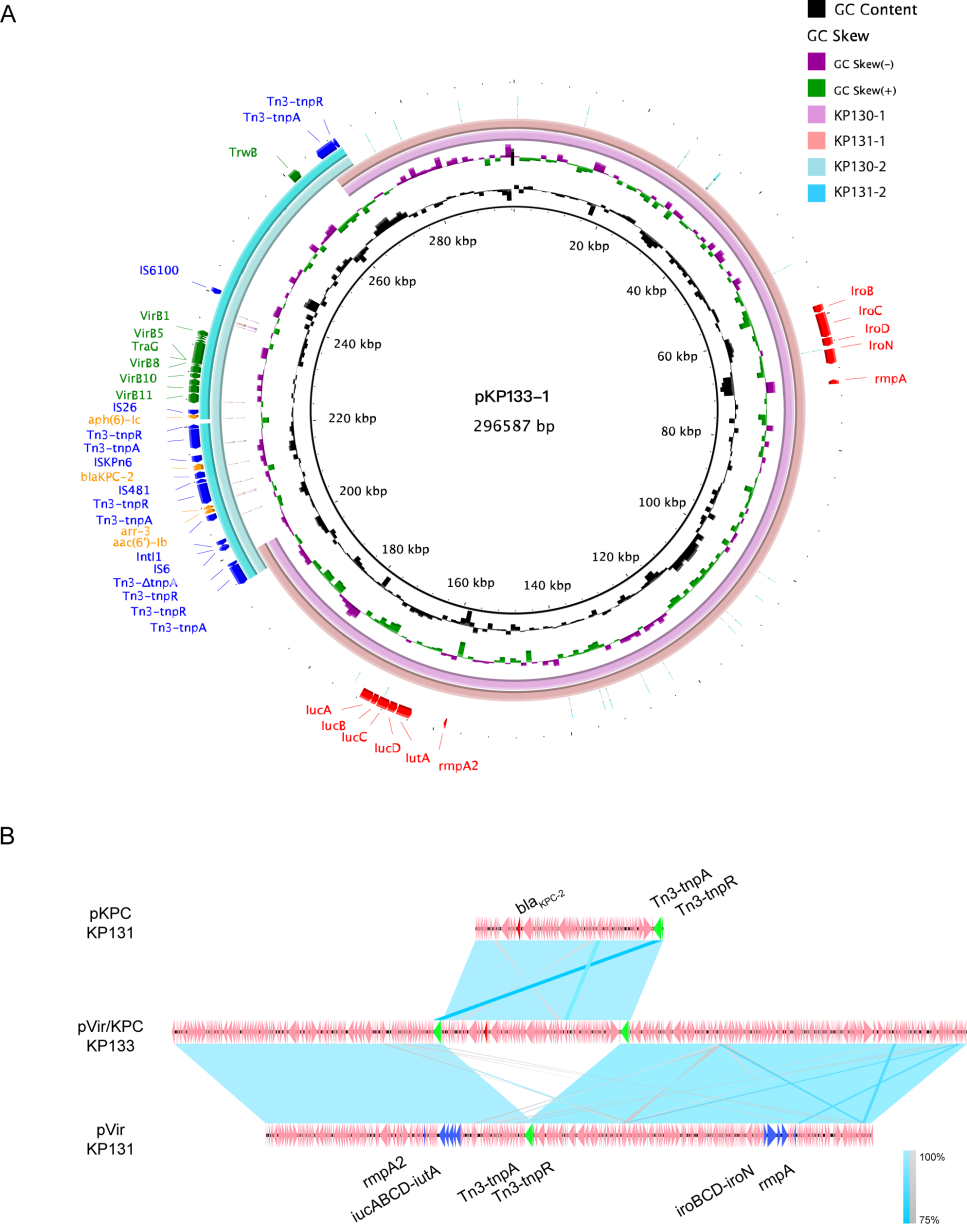
Comparative analysis of pVir/KPC fusion plasmids and other related plasmids. (**A**) Comparative analysis of pVir, pKPC, and pVir/KPC plasmids by BLAST Ring Image Generator (BRIG). (**B**) Alignments of pVir, pKPC, and pVir/KPC plasmids by Easyfig.

BLASTn analysis of pVir plasmids revealed that pKP130-1 shared 100% identity and 100% coverage with pKP131-1, but shared 77% coverage with pKP133-1. The KPC plasmids pKP130-2 and pKP131-2 have shown reasonable similarity with the pKP133-1 plasmid, with 98% and 100% identity, respectively, and coverage of both of about 24%. Comparative genome analysis revealed that pVir/KPC (pKP133-1) was formed by the fusion of the pVir (pKP130-1 or pKP131-1) and the pKPC (pKP130-2 or pKP130-2) plasmids (Fig. 3A). The homologous fusion regions of the pVir and pKPC plasmids contained the Tn3 transposon (Fig. 3B) suggesting that the Tn3 transposon is possibly involved in the fusion of the two plasmids. We also observed that pKP131-2 was formed from the fusion of the pKPC (pKP130-2) and the pCol plasmids (pKP130-3; Fig. S2).

### Mobilization of virulence plasmids mediated by Tn3

**FIG 4:**
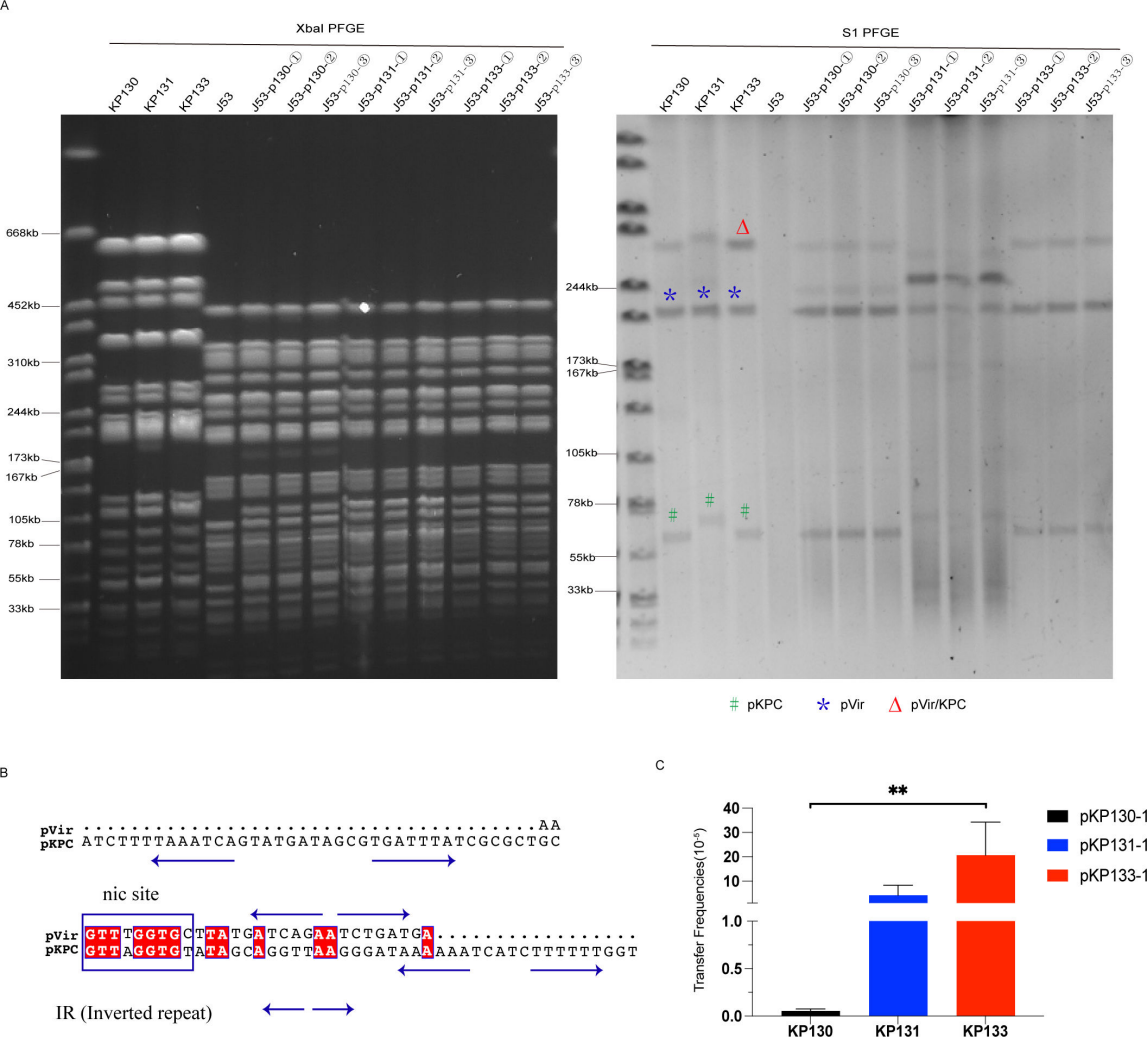
Transfer of virulence plasmids among ST268-CR-hvKp isolates. (**A**) Pulsed-field gel electrophoresis (PFGE) and S1-PFGE. *pVir, #pKPC, ∆pVir/KPC. (**B**) *oriT* sequences of pVir and pKPC plasmids. The alignment was generated with the MUSCLE sequence alignment tool. (C) Transfer frequencies of virulence plasmids of KP130, KP131, and KP133.

Our previous study showed that non-conjugative virulence plasmid can be mobilized by the conjugative IncFII-type KPC plasmid because of the similar *oriT* site (6). Whether the IncN/IncU-type plasmid, with a complete conjugal transfer region, had the ability to mobilize the non-conjugative virulence plasmid needs to be further determined. We successfully conjugated KP130, KP131, and KP133 with sodium azide-resistant J53 and obtained the resulting transconjugants (Fig. 4A). Considering that the three transconjugants of each isolate had identical plasmid profiles [as determined from the S1 nuclease pulsed-field gel electrophoresis (S1-PFGE) assays], one transconjugant from each isolate was chosen for further analysis. The complete genome analysis of the selected transconjugants confirmed that the pVir plasmid was co-transferred to the J53 strain (BioProject: PRJNA899795) with the pKPC plasmid. We observed that the *nic* sites of the pVir plasmids had 77.78% similarity with those from pKPC plasmids (Fig. 4B), suggesting that pVir plasmids could probably have been mobilized by pKPC plasmids. However, the virulence plasmid transfer frequencies have shown significant differences among the KP130, KP131, and KP133 strains (Fig. 4C). Thus, additional mechanisms may also be involved in the non-conjugative pVir plasmid mobilization. Moreover, the p131-1 plasmid of the J53-p131 strain was formed by the fusion of the pVir plasmid with partial sequences of the pKPC plasmid (which included its complete Type IV secretion system; Fig. S3).

**FIG 5:**
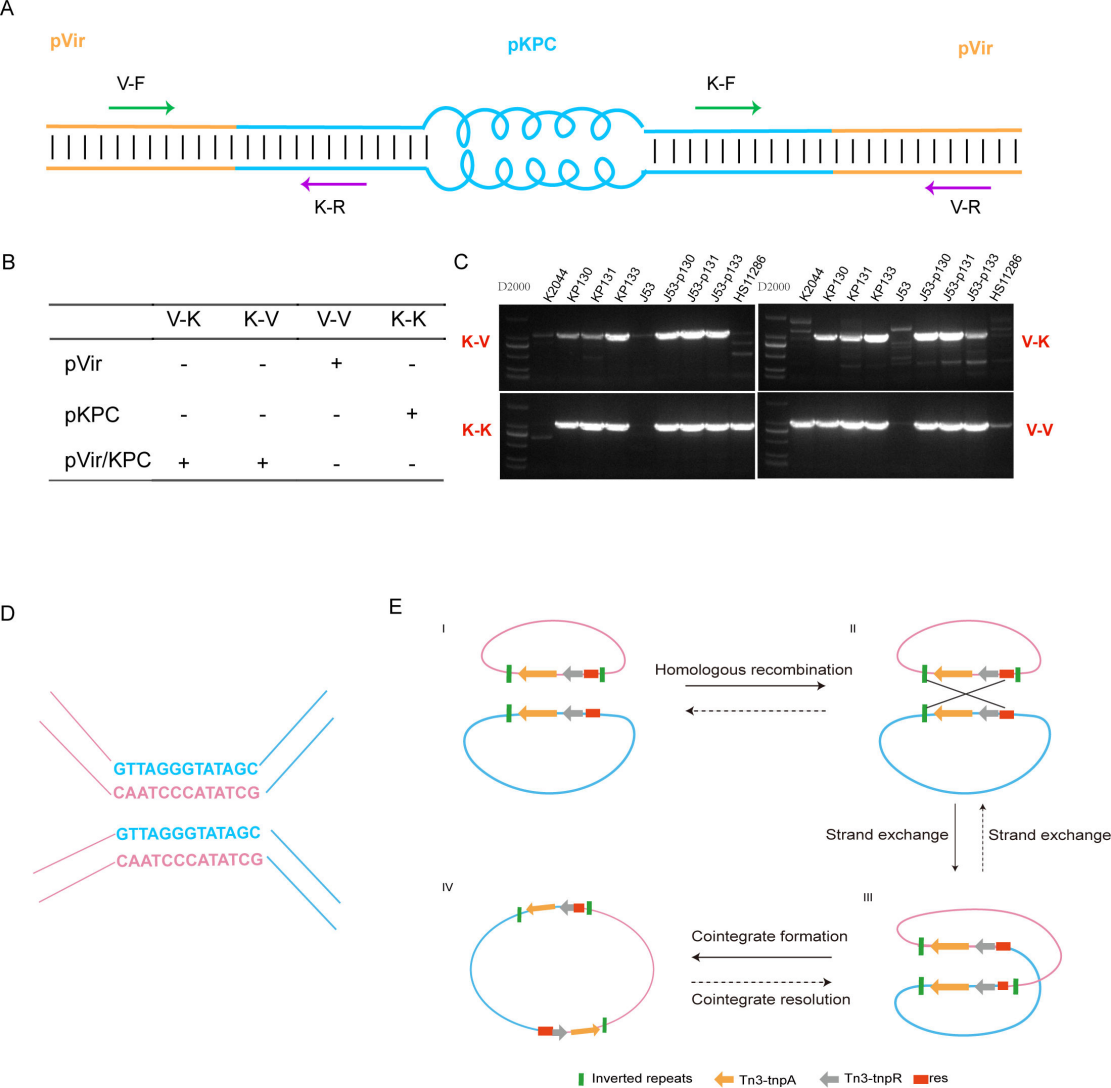
The formation and resolution of pVir/KPC plasmids mediated by Tn3. (**A**) Primers designed for detecting pVir/KPC plasmids. (**B**) Interpretations for the PCR results. V-K: V-F/K-R; K-V: K-F/V-R; V-V: V-F/V-R; K-K: K-F/K-R. (**C**) DNA electrophoretograms for four genes specific for pVir, pKPC, and pVir/KPC plasmids. (**D**) 13 bp sequences involved in fusing with pKPC and pVir plasmids. (**E**) The formation and resolution of pVir/KPC plasmids. pVir fuses with pKPC via Tn3 transposon-based homologous recombination to generate a new pVir/KPC fusion plasmid, and the cointegrate is further resolved by resolvase-mediated site-specific recombination between the duplicated copies of the transposon resolution site.

Interestingly, according to the S1-PFGE results, the pVir/KPC fusion plasmid 290 KB band was found not only in KP133 but also in KP130 and KP131 (Fig. 4A). Likewise, the KP133 strain also exhibited pVir (220 Kb) and pKPC (80 Kb) bands (Fig. 4A). However, these observations are not consistent with the complete genome sequencing results (Table 1). Next, as indicated in Fig. 5A, we designed four specific primer sets (V-K, K-V, V-V, and K-K) to target the sequences of the fusion region between the pVir and pKPC plasmids. If both V-K and K-V are positive, they will indicate the existence of pVir/KPC fusion plasmids in the tested strains. Additional explanations are given in Fig. 5B. Interestingly, the pVir/KPC fusion plasmid was found to be present in the KP130 and KP131 strains, which also carried the pVir and pKPC plasmids (Fig. 5C). Likewise, KP133 carried not only the pVir/KPC plasmid but also the pVir and pKPC plasmids (Fig. 5C). The same observation was obtained in three transconjugants (J53-p130, J53-p131, and J53-p133; Fig. 5C). Taken together, the pVir/KPC, pVir, and pKPC plasmids were simultaneously found in these isolates. However, the pVir/KPC, pVir, and pKPC plasmids could not maintain stable replication due to their incompatibility.

We further dissected the sequences of the Tn3 transposon homologous region and found that the fusion sites could be targeted to 13 bp sequences (Fig. 5D). These sequences were also the resolution site (*res*) located inside the Tn3 transposon, responsible for the cointegration resolution of the transposition process. Based on these observations, we propose that pVir fuses with pKPC via Tn3-based homologous recombination to generate a new pVir/KPC fusion plasmid. Cointegration is further resolved between the duplicated copies of the transposon resolution site by resolvase-mediated site-specific recombination (Fig. 5E).

### Transmission of virulence and carbapenem resistance

Next, we investigated the changes in virulence and resistance potential of the recipient strains J53-p130, J53-p131, and J53-p133 after the acquisition of the plasmids conferring these properties. The J53 strain was sensitive to all tested antibiotics, while the transconjugants (J53-p130, J53-p131, and J53-p133) acquired resistance to cephems, β-lactam combination agents, and carbapenems. Nevertheless, they remained sensitive to levofloxacin, gentamicin, chloramphenicol, trimethoprim/sulphamethoxazole, nitrofurantoin, and tigecycline (Table 2).

In comparison with the parental J53 strain, the J53-p130, J53-p131, and J53-p133 transconjugants have shown clearly higher siderophore production levels, while their larvae-validated virulence levels were not increased (Fig. S4). Other virulence phenotypes, such as serum resistance and viscosity, were also not significantly changed (Fig. S4).

## DISCUSSION

An epidemic of hypervirulent and carbapenem-resistant *K. pneumoniae* has a higher mortality rate and is a greater public health challenge than an epidemic of the *K. pneumoniae* CRKP or hvKp strains (9, 10, 19). The mobilization of plasmids aggravates the emergence of such hypervirulent and carbapenem-resistant pathogens, regardless of the evolutionary pathways. Our previous studies simulated the evolutionary path of the CR-hvKp strains and found that the CR-hvKp strain could not simultaneously maintain hypervirulence and carbapenem resistance due to the *rfaH* mutation (6). *K. pneumoniae* in the CR-hvKp pattern have been reported, such as ST23-CR-hvKp (20 – 22), but little is known whether they exhibited both phenotypes: hypervirulence and carbapenem resistance. In this study, we collected three ST268-K20 *K. pneumoniae* strains (CR-hvKp pattern), which were successfully isolated from patients of the same hospital ward, and simultaneously carried the pLVPK-like virulence and *bla*_KPC-2_-positive plasmids.

Most hvKP strains have either a K1 or K2 bacterial capsule type (23). Although the K20-type has been reported as hypervirulent (24), there are no previous reports on the evolution, virulence, and resistance of the ST268-CR-hvKp strain. We dissected the genetic relatedness of ST268 adjacent genomes and found that ST268 was located between CG15 and CG23. CG15 is always associated with antibiotic resistance, while CG23 is the most common hvKp type (12, 25). The mean virulence and mean resistance scores were also intermediate between the two groups. The SNP-based phylogenetic tree showed clear evidence that ST268 had a close relationship with hypervirulent ST65 isolates rather than CG15 MDR isolates. This finding suggests that ST268-CR-hvKp isolates first became hypervirulent and then acquired the resistance plasmid.

HvKp isolates usually produce hyperviscous mucoid capsules on the bacterial surface; nevertheless, there is no clear understanding of their genetic basis and contribution to disease pathology (26). Although the KP130, KP131, and KP133 *K. pneumoniae* strains exhibited hypervirulence, they lacked a hyperviscous mucoid phenotype and capsule overproduction. This could be due to mutations in the *rmpA* and *rmpA2* genes, which are involved in the biosynthesis of CPS. There is extensive evidence showing that *K. pneumoniae* strains carrying a virulence plasmid do not necessarily display a hypermucoviscous phenotype, and a hypermucoviscous phenotype may not also correlate with a hypervirulent phenotype of this organism (27, 28). Among other factors, the ST268-CR-hvKp strain hypervirulence might be due to high levels of siderophore production. Siderophores, like aerobactin (encoded by *iutA-iucABCD*), are also critical virulence factors, enabling bacterial iron acquisition needed by hvKp strains, to proliferate infection (29). Moreover, ST268-CR-hvKp isolates also exhibited high resistance levels to many antibiotics. Therefore, ST268-CR-hvKp isolates were hypervirulent and carbapenem-resistant, while other *K. pneumoniae* strains in the CR-hvKp pattern may not exhibit carbapenem resistance and hypervirulence simultaneously. These findings indicate that ST268-K20 *K. pneumoniae* is more likely to evolve into hypervirulent and carbapenem-resistant strains compared with other types in the CR-hvKp pattern.

A non-conjugative plasmid harboring a mimic *oriT* sequence of conjugative plasmid can be mobilized by the *in-trans* activity of this conjugative plasmid (30). This *in-trans* relaxase mechanism of plasmid mobilization has also been confirmed in *K. pneumoniae* and *Staphylococcus aureus* (7, 31). In this study, we provided evidence that the conjugative IncN/U plasmid may also mobilize the pLVPK-like non-conjugative virulence plasmid, enriching our understanding of the transmission and evolution of virulence plasmids. However, significant differences in transfer frequencies were observed among the KP130, KP131, and KP133 strains. The high transfer frequency of the pVir/KPC plasmid of the KP133 strain could be attributed to harboring a complete transfer element, conferring the self-transfer ability to the pKPC/Vir plasmid. We speculate that additional mechanisms affecting pVir plasmid transfer into KP130 and KP131 strains may exist. Notably, complete genome sequencing and S1-PFGE have produced conflicting results. Thus, four primer sets have been further designed, and electrophoretic results confirmed the presence of the pVir, pKPC, and pVir/KPC plasmids in three ST268 CR-hvKp strains. Complete genome sequencing may have some constraints on low-copy plasmids. These plasmids were unstable in the host bacteria due to the plasmid incompatibility (32). Thus, genomic sequencing failed to identify sequence differences among the plasmids carried by the KP130, KP131, and KP133 strains. Taken together, pVir plasmids can be transferred by Tn3-mediated fusion with self-transferable pKPC plasmid medicated by Tn3, or can also be directly mobilized by pKPC plasmids. Moreover, fusions of the pVir plasmid with specific sequences of the pKPC plasmid (including the complete transfer region) in J53-p131 transconjugants may confer the higher transfer frequencies of pVir plasmids of KP131, in comparison with those of KP130 strains.

Plasmid conjugation is one of the basic methods of horizontal gene transfer, a process involved in the DNA exchange between neighboring bacteria. Although conjugation transfer provides a reasonable explanation for the acquisition of resistance and virulence, it can also cause gene rearrangements. The Tn3 transposon, which was found both in the pVir and the pKPC plasmids, mediates the formation of the pVir/KPC fusion plasmid by homologous recombination. This is the most common mechanism leading to the integration or fusion of two plasmids. It is becoming increasingly common for virulence plasmids to recombine with other plasmids. There can be mosaic virulence plasmids that encode both resistance and virulence genes, or conjugative virulence plasmids that enhance virulence gene transfer efficiency (32 – 34). The Tn3 transposon family encodes a DNA site-specific recombinase (or “resolvase”) that resolves the cointegrate intermediate. Recombination occurs at specific transposon sites, the “internal recombination site” (IRS) or the “resolution site” (*res*) (15). Considering plasmid fusion at the *res* site and the simultaneous coexistence of pVir/KPC, pVir, and pKPC plasmids, it is reasonable to infer that pVir/KPC plasmid fusion and dissociation occur dynamically. The pVir plasmid could fuse with the pKPC plasmid by homologous recombination to generate a new fusion plasmid. The cointegrate is further resolved by resolvase-mediated site-specific recombination between the duplicated copies of the transposon resolution site. Recombination events facilitate plasmid evolution and confer adaptation advantages to complex and volatile environments. The transfer of resistance and virulence plasmids promotes the emergence and dissemination of CR-hvKp or hv-CRKP strains, posing challenging problems for public health and healthcare systems.

Our study had limitations that should be taken into account while interpreting these findings. First, there were a limited number of strains involved in this study. Second, the complexity of clinical strains cannot be avoided. Our study uncovers a novel mechanism of CR-hvKp transmission and cannot be generalized for all CR-hvKp strains. These limitations could motivate future research on more potential mechanisms of CR-hvKp transmission.

### Conclusions

The pVir plasmid can be transferred via mobilization by the conjugative IncN/U-type pKPC plasmid, as well as by fusing with the conjugative pKPC plasmid (mediated by the Tn3 transposon) to be self-transmissible. The involvement of transposons greatly enhances the efficiency of non-conjugative virulence plasmids transmission. We must be vigilant to emerging transposon-mediated hypervirulent and carbapenem-resistant pathogens, as transposons are ubiquitous in *Enterobacteriaceae* and contribute to the spread of resistance and virulence genes.

## MATERIALS AND METHODS

### Bacterial strains and microbiological characteristics

The *K. pneumoniae* KP130, KP131, and KP133 strains were isolated from clinical specimens from different patients in the same ward at a hospital in Zhejiang involved in our previous retrospective multicenter study (6). Virulence-associated genes (*iucA, iroB, rmpA*, and *rmpA2*) and carbapenem resistance genes were first amplified and sequenced. The antimicrobial susceptibilities to common antibiotics were determined by the microdilution method. The results were interpreted according to the Clinical and Laboratory Standards Institute (CLSI M100) (35).

### Pulsed-field gel electrophoresis and S1 nuclease pulsed-field gel electrophoresis

The PFGE methodology was used to analyze the genetic relationship among the KP130, KP131, KP133 *K. pneumoniae* strains, recipient *Escherichia coli* J53, and their transconjugants. These isolates were digested by XbaI endonuclease (Takara, Dalian, China) and analyzed by PFGE according to a previous study (36). The S1-PFGE methodology was used to determine the numbers and molecular weight of the plasmids carried by the isolates involved in this study. The relevant isolates digested with S1 nuclease (Takara, Dalian, China) were subjected to PFGE as described above.

### Complete genome sequencing and bioinformatic analysis

After bacterial DNA isolation, the genomes of the KP130, KP131, and KP133 *K. pneumoniae* strains, as well as their transconjugants, were sequenced using the Illumina NovaSeq and MinION platforms. *De novo* assembly was performed using HGAP and CANU using default settings (37, 38). Assembly errors were corrected using Pilon (39), prior to obtaining the complete genome. To screen ST268-related genome sequences, we collected 1,165 publicly available *K. pneumoniae* genomes from the GenBank database (Supplementary Data 1). *Kleborate* was used to determine the sequence type, K_locus, antimicrobial resistance genes, and virulence genes of the strains (http://github.com/katholt/Kleborate). The circular maps for plasmid genome comparison were generated using the BLAST Ring Image Generator (BRIG) software (http://sourceforge.net/projects/brig) (40). A comparison of the fusion regions of related plasmid genomes was performed using Easyfig software (https://mjsull.github.io/Easyfig/) (41). *oriT* sequences and transfer elements were predicted using oriTfinder’s default parameters (42).

### Phylogenic analysis

The clusters of ST268 adjacent genomes (Supplementary Data 2) were evaluated using the goeBURST software. The phylogenic analysis of related genomes was performed by Parsnp and visualized in iTOL. The virulence scores and resistance scores were calculated according to the algorithm described in a previous study (25).

### Confirmation of virulence phenotypes

A series of virulence phenotypic assays were conducted as described in a previous study with some modifications (6, 8). The serum resistance assay was performed by mixing mid-log phase bacteria with serum from healthy people (at a 1:3 ratio), followed by a 3-h incubation at 37°C. The survival percentage of each strain was plotted against the incubation period to determine the serum resistance. The hypermucoviscosity was determined by measuring the absorbance at 600 nm of supernatants after centrifugation at 10,000 *g* for 30 s. The siderophore production was confirmed by two methods: agar plates containing Chrome Azurol S and quantification of the siderophore production by Siderophore units (Su). The uronic acid content was quantified to evaluate CPS production. The virulence levels were assayed both in the mice and *Galleria mellonella* larvae infection models. The bacterial inoculum employed in mice was adjusted to 5 × 10^6^ colony-forming units (CFU). The bacterial inoculum of the KP130, KP131, and KP133 strains employed in larvae was adjusted to 1 × 10^5^ CFU. In transconjugants, it was increased to 1 × 10^6^ CFU in mice and 1 × 10^7^ CFU in *G. mellonella*. All experiments were repeated at least three times except the mice infection experiments, which were repeated twice.

### Plasmid conjugation assays

The plasmid conjugation assays were conducted to determine whether the virulence plasmids of the *K. pneumoniae* KP130, KP131, and KP133 strains could be transferred to other strains, conferring virulence and resistance. KP130, KP131, and KP133 with tellurite resistance were used as donors, while sodium azide-resistant J53 was used as the recipient. We cultured both donors and recipients to the logarithmic phase at 37°C. After mixing 100 µL of donor cells and 400 µL of recipient cells, an LB agar plate was inoculated and incubated at 37°C for 16–18 h. Transconjugants were selected with potassium tellurite (3 µg/mL) and sodium azide (150 µg/mL). The transconjugants were further amplified by PCR using *iucA* and *bla*_KPC-2_ primers. Three transconjugants of each strain were chosen for PFGE and S1-PFGE analysis, as described above. Next, the complete genome of these transconjugants was sequenced to determine the numbers and molecular weights of the plasmids, as well as to detect any recombination events during the transmission process.

### Statistical analysis

GraphPad Prism 9 (GraphPad Software, San Diego, CA, USA) was used to assess the statistical significance of the data with the Student’s *t*-test and the log-rank test.

## Data Availability

The original data that support the conclusions of this article are presented in the article or in the supplemental information. The complete sequences of strains used in this study were submitted to the GenBank database (Bioproject: PRJNA833743, PRJNA899795).

## SUPPLEMENTAL MATERIAL

Supplemental file 1 spectrum.03038-23-s0001.docx Fig. S1 to S4 and Table S1.

Supplemental file 2 spectrum.03038-23-s0002.xlsx Data Sets S1 and S2.

ASM does not own the copyrights to Supplemental Material that may be linked to, or accessed through, an article. The authors have granted ASM a non-exclusive, world-wide license to publish the Supplemental Material files. Please contact the corresponding author directly for reuse.
