# Supplementary material for: Involvement of Tn3 transposon in formation and transmission of hypervirulent and carbapenem-resistant Klebsiella pneumoniae
Source: Microbiol Spectr. 2023 Nov 20;11(6):e03038-23. doi: 10.1128/spectrum.03038-23 (PMC10714771; doi:10.1128/spectrum.03038-23)
Supplement: Supplemental file 1 — Fig. S1 to S4 and Table S1. [file spectrum.03038-23-s0001.docx]

**Supplementary Materials**

**
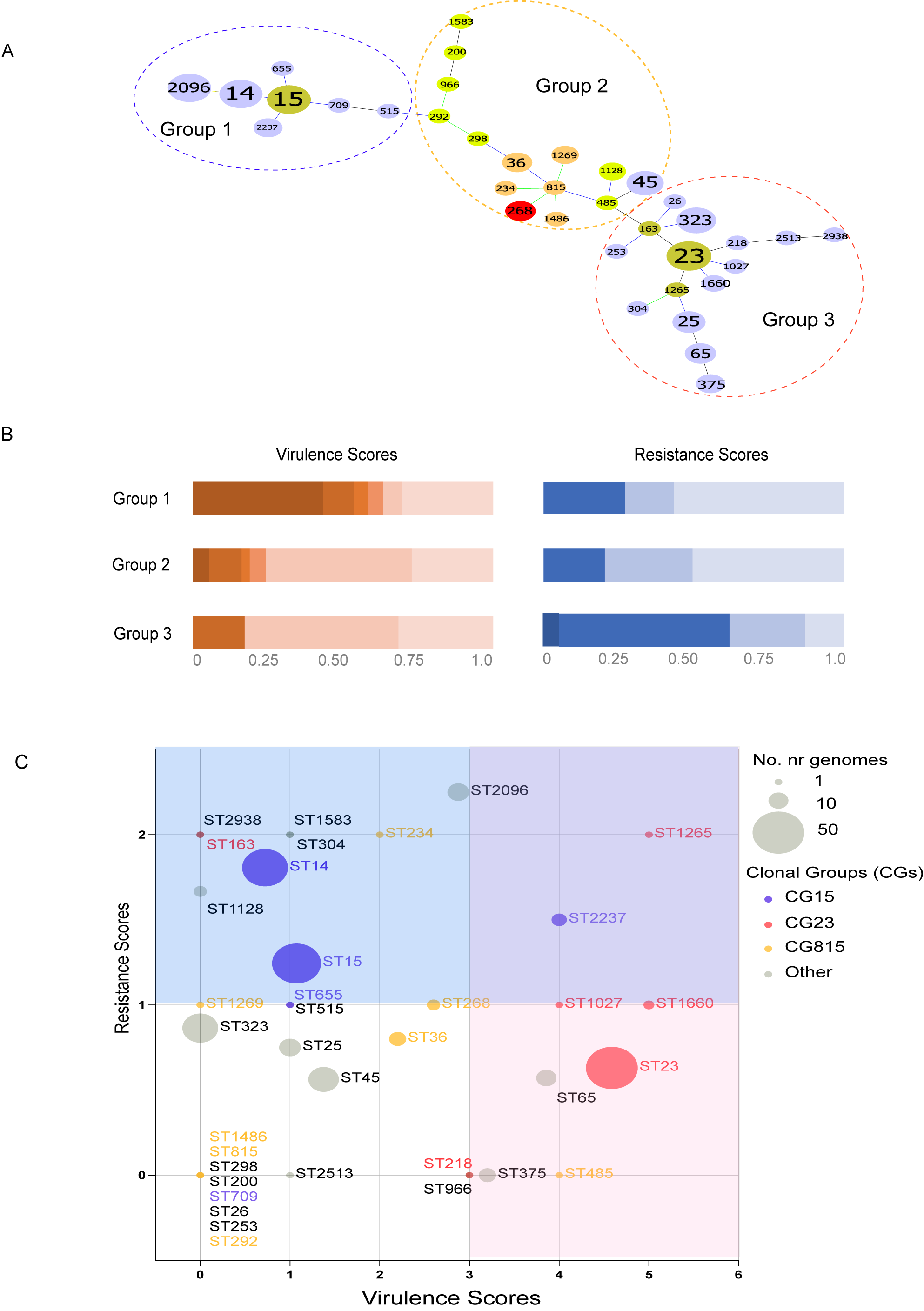
Fig. S1: Genetic relationship and characteristics of ST268 and adjacent genomes.** The data shown summarizes *Kleborate* results for 228 ST268-related *Klebsiella* genomes (Supplementary Data 1). (A) Genetic clusters were determined by the goeBURST software. (B) Mean virulence and resistance scores among different genetic groups. (C) Mean virulence and resistance scores grouped by STs. Each circle represents a single ST; size indicates the number of genomes (as per inset legend); colour indicates groups per inset legend.

**
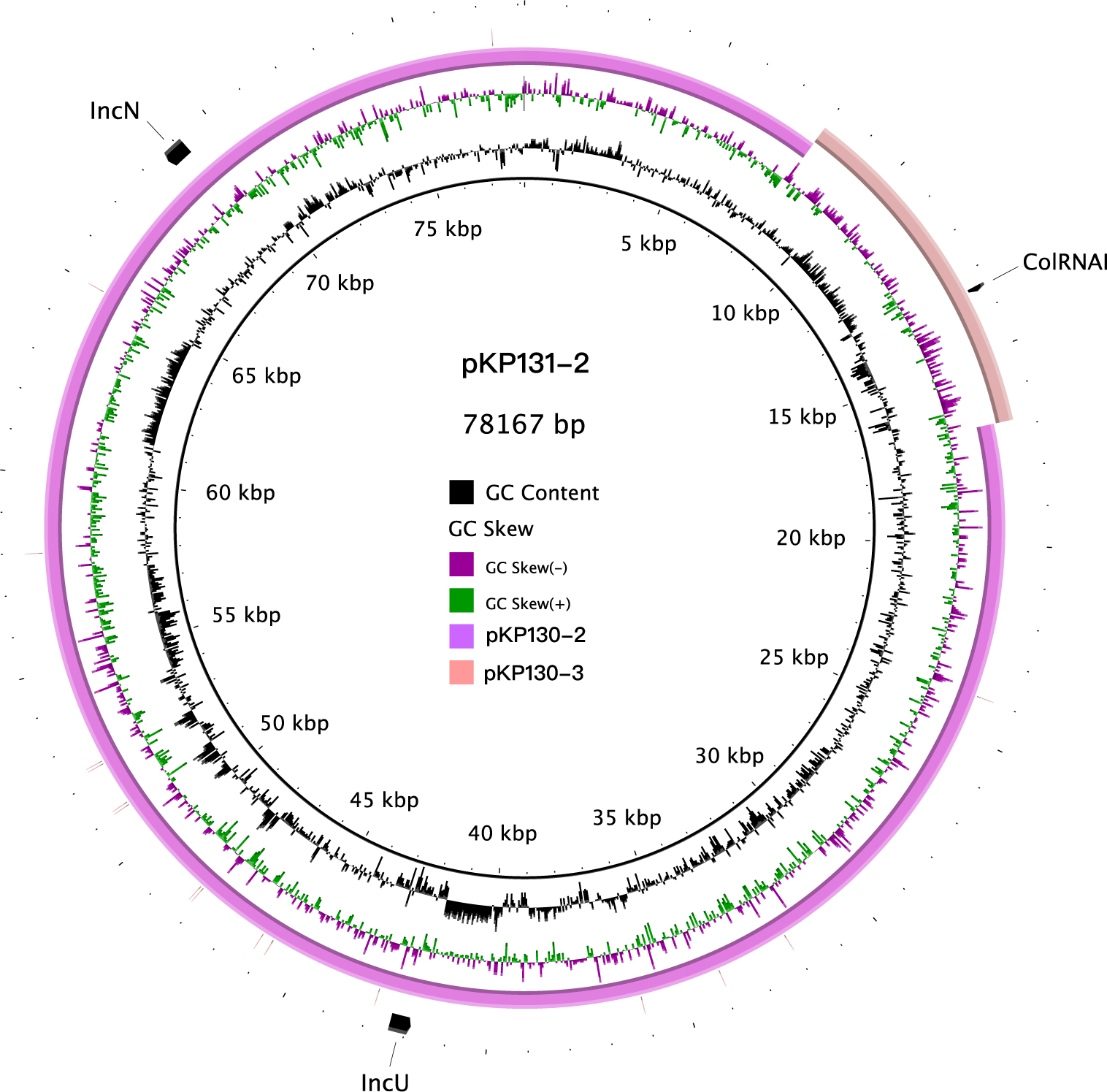
**

**Fig. S2: Comparative analysis of pKP131-2, pKP130-2, and pKP130-3.**

**
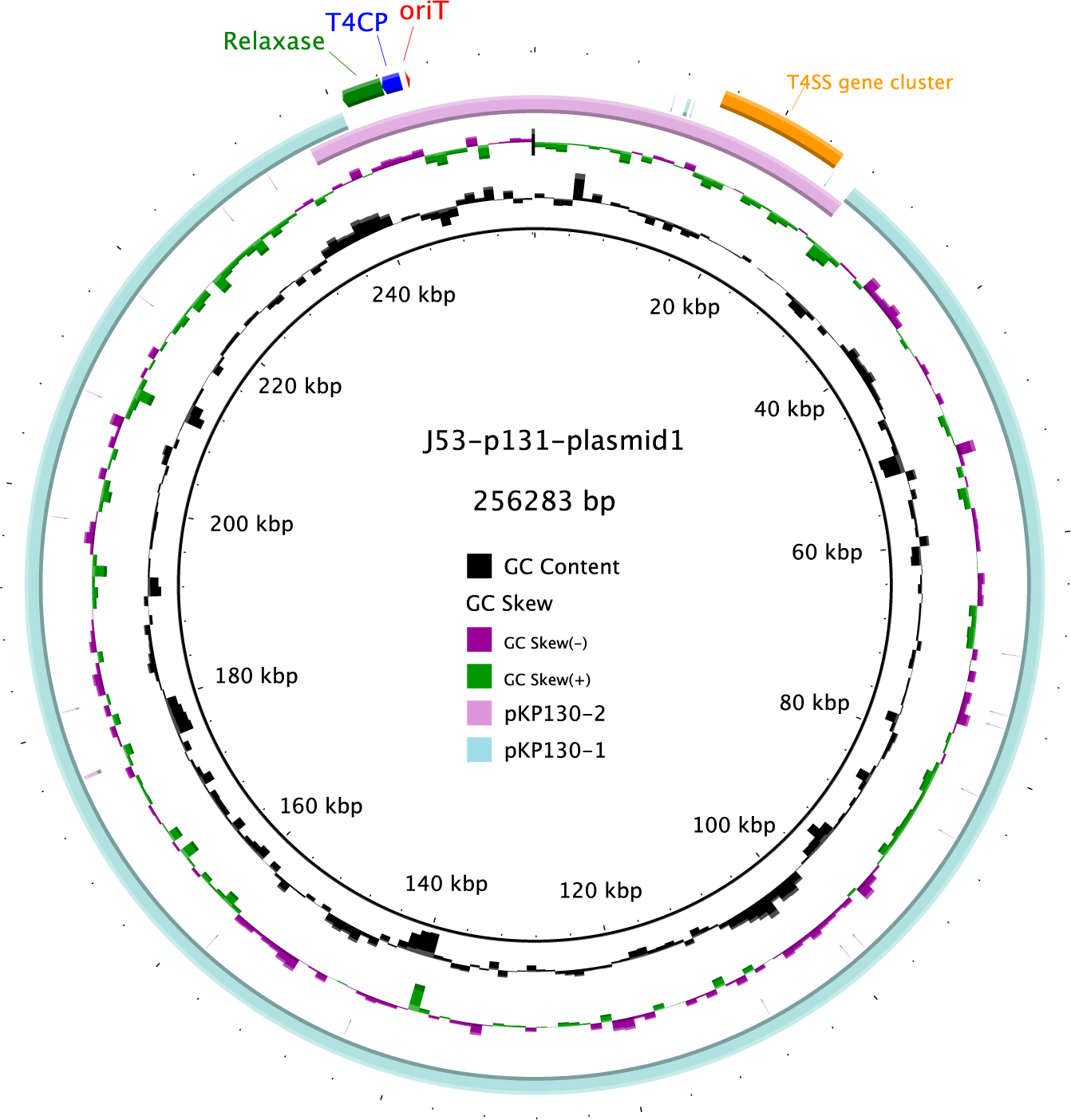
**

**Fig. S3: Comparative analysis of J53-p131-1, pKP130-1, and pKP130-2.**

**
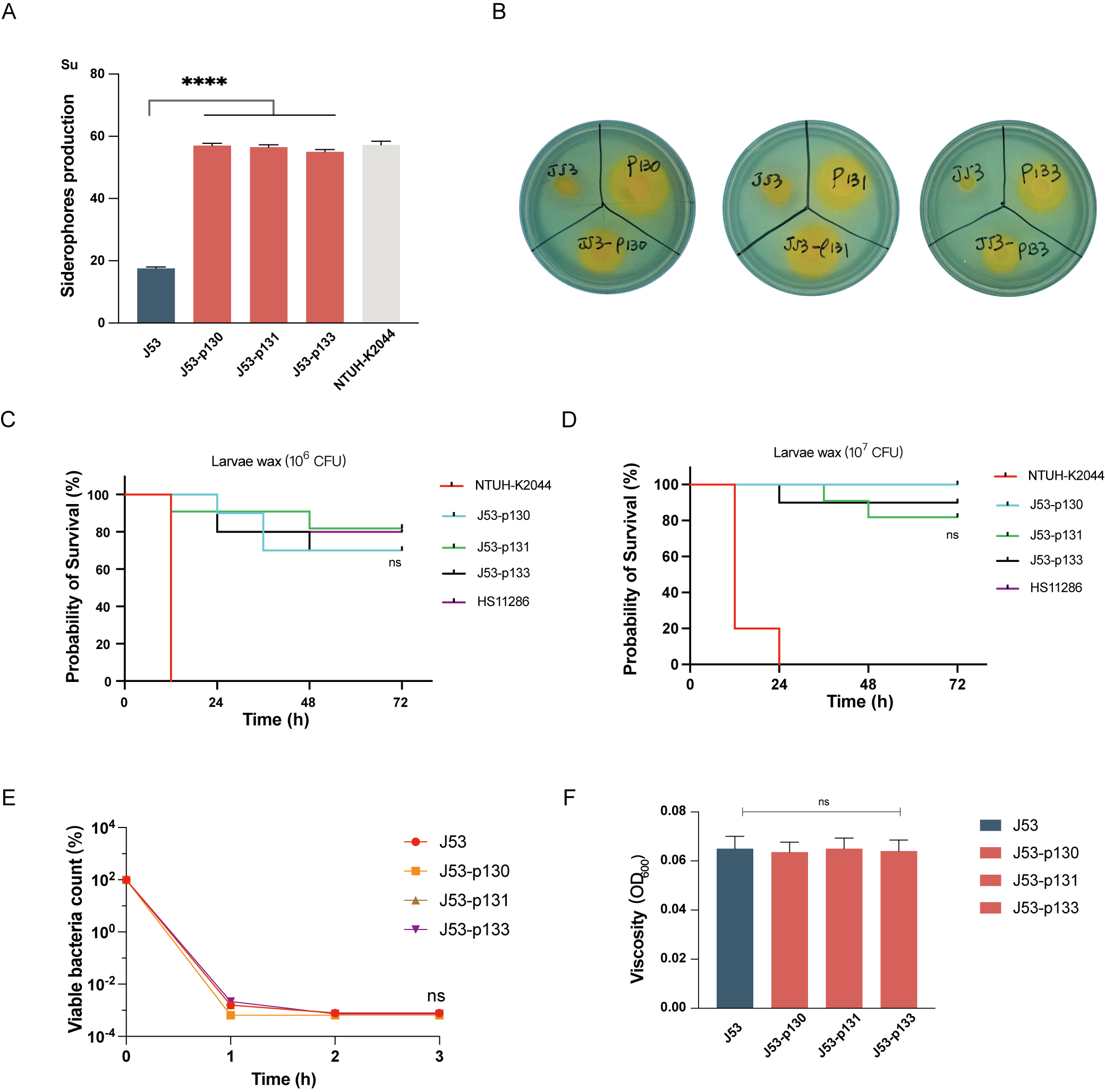
**

**Fig. S4: Virulence-associated phenotypic tests for transconjugants.** (A) Quantification of siderophore production. (B) Siderophores production (CAS agar assays). (C) Survival curves of infected larvae wax (1×10^6^ CFU). (D) Survival curves of infected larvae wax (1×10^7^CFU). (E) Serum resistance. (F) Viscosity. An unpaired two-sided Student’s t-test was performed for mucoviscosity, uronic acid, siderophores production analysis. A log-rank (Mantel–Cox) test was performed for the survival curves (****P< 0.0001, ns: not significant).

**Table S1 Primers used in this study**

| **Name** | **Sequences (5’-3’)** |
| --- | --- |
| **For screening transconjugants** | |
| iucA-F | GCTTATTTCTCCCCAACCC |
| iucA-R | TCAGCCCTTTAGCGACAAG |
| KPC-F | CTGTCTTGTCTCTCATGGCC |
| KPC-R | CCTCGCTGTGCTTGTCATCC |
| **For detecting of the pVir/KPC plasmids** | |
| V-F | ACTTGGCAATTTGTTCGCTGTT |
| V-R | GCAAGTCGAAATCACCCCGTT |
| K-F | AGCCTGCTCATCGGACAGG |
| K-R | TTGGAGCGGTAAGACCAACG |
